# Hearing Performance and Soft-Tissue Outcomes of Minimally Invasive Ponto Surgery and Local Anesthesia in Children with Unilateral Craniofacial Malformation

**DOI:** 10.1055/s-0044-1788910

**Published:** 2025-01-27

**Authors:** Andrea Caruso Leone, Arthur Menino Castilho, Fabiana Danieli, Daniela Bortoloti Calil, Katia de Almeida

**Affiliations:** 1 Postgraduate Program in Human Communication Health, School of Medical Sciences, Santa Casa de São Paulo, São Paulo, SP, Brazil; 2 Department of Otorhinolaryngology, Head and Neck Surgery, School of Medical Sciences, Universidade Estadual de Campinas (UNICAMP), Campinas, SP, Brazil; 3 Department of Scientific and Clinical Research, Oticon Medical, São Paulo, SP, Brazil; 4 University Hospital, School of Medical Sciences, Universidade Estadual de Campinas (UNICAMP), Campinas, SP, Brazil; 5 School of Medical Sciences, Santa Casa de São Paulo, São Paulo, SP, Brazil

**Keywords:** bone conduction, minimally invasive, bone drilling, craniofacial abnormalities, bone-anchored prosthesis, children

## Abstract

**Introduction** Minimally invasive Ponto surgery (MIPS) enables the installation of percutaneous bone-anchored hearing implants (BAHIs) with a drill guide through a hole punch incision. Despite being well established for adults, there is a lack of studies in the literature regarding its use in pediatric patients.

**Objective** The aim of the present study was to investigate the hearing performance and soft-tissue outcomes of the use of MIPS under local anesthesia in children with unilateral craniofacial malformation (UCM).

**Methods** The study used a retrospective cohort design. Nine subjects with UCM, aged between 6.5 and 17.1 (median = 12) years, who underwent the MIPS procedure under local anesthesia were included. Surgical procedure, intra, and postoperative complications were investigated. Speech recognition thresholds in quiet (SRTQ) and in noise (SRTN), daily use, satisfaction, and perceptual listening effort of the subjects were assessed after 4 months of postoperative follow-up.

**Results** It was possible to perform MIPS under local anesthesia in 8 of 9 subjects, with no intraoperative complications. One subject (11.11%) showed adverse skin reactions during a mean follow-up period of 11.4 months with MIPS. Speech recognition thresholds in quiet, SRTN, and subjective listening effort scores significantly decreased with the use of BAHI. The subjects were overall satisfied with the device and using it 8.2 hours/day, on average.

**Conclusion** Under local anesthesia, MIPS showed to be a viable option for BAHI installation in children with UCM. The hearing performance of the subjects improved, and they were globally satisfied with the device. Soft-tissue complications were minimal, and our results are comparable to those reported in the literature for adults.

## Introduction

The percutaneous bone-anchored hearing system (BAHS) is a valuable treatment option for patients with conductive hearing loss, mixed hearing loss, or single-sided deafness. It is comprised of a titanium implant inserted into the calvarium and integrated into the skull from the growth of bone tissue over its titanium surface, an abutment fixed to the implant through the skin, and an external sound processor attached to the abutment, thereby allowing direct bone conduction, that is, sound energy transmission from the skull vibrations to the cochlea, which ultimately results in wave propagation along the basilar membrane and stimulation of the cochlear nerve. 1

The BAHS was first introduced in 1977, 2 and the U.S. Food and Drug Administration (FDA) approved the surgery in children with ages over 5 years old in 1999. 3 The surgical technique was initially performed in two-stages in both adults and children, intended for implant installation and abutment-implant attachment associated to the soft-tissue reduction, both performed separately. Technological advances in the BAHS design, such as wider diameter implants and different abutment length options, enabled the performance of installation mostly in one-stage, with soft-tissue preservation. It reduced the postoperative complications, adverse skin reactions and implant failure, and thereby becoming the gold standard in adult patients. 4 Aiming to reduce even more the invasiveness of the surgical technique, the first minimally invasive punch-only surgical technique was introduced, 5 allowing to install the BAHS implant with the use of a drill guide through a hole punch incision. The use of this surgical technique showed additional benefits, such as shorter operative times, faster healing, and less soft-tissue reaction compared with the standard linear incision technique in adults. 6 Although it is one of the least invasive procedures available to install BAHS implants, there is a lack of studies in the literature regarding its results among the pediatric population. A recent systematic review 4 showed that new surgical techniques for percutaneous BAHS implant installation are promising for pediatric patients and claimed for new studies on the topic. It concluded that one-stage surgery and soft-tissue preservation do not provide higher implant loss or adverse skin reactions to pediatric patients and then, new techniques could additionally benefit this population.

In 2021, French et al. 7 performed a retrospective study on minimally invasive Ponto surgery (MIPS) in 14 pediatric patients and found similar results to those reported for adults. To the best of our knowledge, this is the only study involving minimally invasive punch-only technique to install a BAHS implant specifically in pediatric patients, but anesthetics procedures were not investigated. Sardiwalla et al. 8 reported that MIPS under local anesthesia may be performed as an outpatient procedure, outside of the main operating room, leading to a further reduction in surgical time, staffing, and running costs, and allowing a higher patient throughput. Then, the use of MIPS associated to local anesthesia could also reduce the exposure to general anesthesia and allow earlier (re)habilitation process in pediatric patients.

Based on this, the aim of the present study was to investigate the hearing performance and soft-tissue outcomes of the MIPS procedure under local anesthesia to install the BAHS implant in children with unilateral craniofacial malformation (UCM).

## Methods

### Study Design

The study used a retrospective cohort design. The inclusion criteria comprised subjects aged up to 17 years old, with UCM, who underwent a BAHS surgery with the one-stage MIPS and local anesthesia (2% lidocaine with 1:200,000 epinephrine). The exclusion criteria comprised subjects who showed up to 4 months of postoperative follow-up, since it was considered the minimal experience period with the bone conduction stimulation needed to perform speech perception tests. The postoperative follow-up period comprised the time between the first abutment fitting and the evaluation.

Surgical procedure, implant and abutment lengths, surgical time, and intraoperative complications (bleeding and implant instability) were investigated. Postoperative complications such as implant loss and revision surgery, as well as soft-tissue outcomes were also investigated during the entire postoperative follow-up period for each subject. Soft-tissue outcomes were scored according to the Holger scale, 9 and it was determined that a Holger score ≥ 2 was representative of adverse skin reactions. 10

All subjects were fitted with the Ponto 3 SuperPower sound processor (Oticon Medical, Smørum, Denmark) and programmed according to the manufacturer's instructions, using the Genie Medical 2016.1 fitting software (Oticon Medical). The gain was calculated by the software according to the desired sensation level-bone conduction (DSL-BC) prescription formula and, finetuned to the subject's preference when required.

The Sentences in Portuguese Test 11 was used to assess the subject's speech recognition in quiet and noise, in unaided and aided conditions, that is, without and with the use of the BAHS, respectively. The test was composed of 60 sentences distributed into 6 lists of 10 phonetically balanced sentences with up to 7 words with lexical/semantic or grammatical meaning, and with similar phonetic content.

**Fig. 1:**
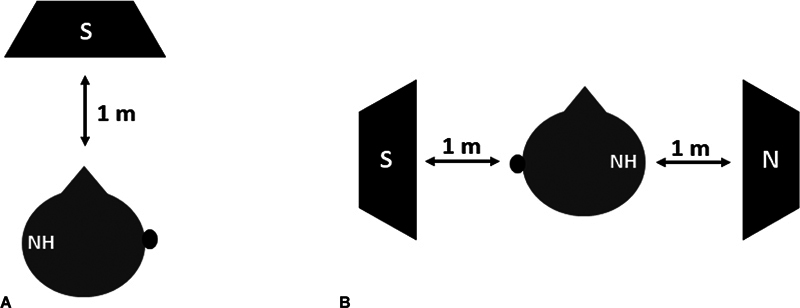
Positioning of the subjects and loudspeakers to the speech recognition test in quiet ( **A** ) and noise ( **B** ) situations. Abbreviations: N, noise, “H, normal hearing; S, sentences. Note: The black circle corresponded to the bone-anchored hearing system (BAHS) device.

The test in quiet was performed using one loudspeaker positioned 1 meter from the patient, in front (0° azimuth, Fig. 1A ). The sentences were first presented at 65 dB SPL, and the presentation level varied according to the subject's response, using an ascending-descending technique. 12 The results were expressed in sentence recognition threshold in quiet (SRTQ), which corresponded to the presentation level (dB SPL) at which the subject correctly repeated 50% of the sentences. The test in noise was carried out using 2 loudspeakers, positioned 1 meter from the patient, in the S90N90 position: the sentences were presented in the implanted ear, and the noise was presented in the contralateral ear ( Fig. 1B ). In this condition, the noise was fixed at 65 dB, and the presentation level of the sentences varied according to the subject's response. The results were expressed in sentence recognition in noise (SRTN), which corresponded to the signal-to-noise level (dB SNR) at which the subject correctly repeated 50% of the sentences. The SRTQ and SRTN were then compared between unaided and aided (BAHS) conditions in all subjects.

**Fig. 2:**
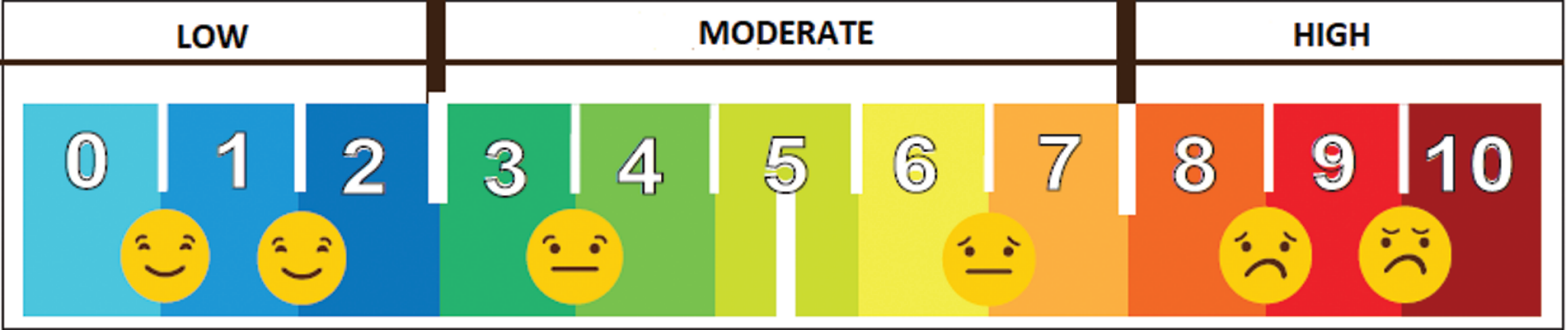
Visual analogue scale used to score the subjective listening effort. Scores 0–2, 3–7 and 8–10 corresponded to low, moderate, and high listening effort perceived by the subjects, respectively.

The subjective listening effort of the subjects was also investigated in both the quiet and noise situations. The subjects scored their perceptual listening effort using a visual-analogue scale ( Fig. 2 ). Scores from 0 to 2, from 3 to 7, and from 8 to 10 corresponded to the low, moderate, and high subjective listening effort categories, respectively. The results were compared between the unaided and aided (BAHS) conditions in all subjects.

All subjects filled the questionnaire Satisfaction with Amplification in Daily Life (SADL) 13 to quantify their overall satisfaction with the device. The subjects who were not capable of answering the questionnaire by themselves had assistance from their parents to complete it. The subject's daily use of the device was objectively assessed using the Genie Medical fitting software, version 2016.1 (Oticon Medical).

The Brazilian Ethics Committee approved this research under protocol number 4.217.145. The legal guardians of all patients gave their written informed consent to participate in this study.

### Data Analysis

The Wilcoxon signed-rank test was used to compare the speech recognition thresholds and subjective listening effort of the subjects, between the unaided and aided conditions. A significant level of 5% was adopted.

## Results

Nine subjects aged between 6.5 and 17.1 (median = 12) years met the inclusion criteria. Table 1 shows the subject demographics. It was possible to perform MIPS under local anesthesia in 8 of 9 subjects (88.9%). For one subject (#S8), the surgical technique was converted to a linear incision with tissue preservation (LITP) due to implant instability and bleeding. Sedation was also administered to this subject. Table 2 shows the surgical and postoperative soft-tissue outcomes of the subjects. The mean postoperative follow-up period was of 11.4 ± 6.7 months. Two subjects (#S4 and #S8) showed adverse skin reactions (Holgers score ≥ 2) during the entire postoperative follow-up period, which were easily solved with local care.

**Table 1:** Subject demographics

Subject		Sex	Age at surgery (years)	Implanted ear	Type of HL (implanted ear)	Degree of HL (implanted ear)	Contralateral ear
1		M	12	R	Mixed	Profound	NH
2		F	6.5	R	Conductive	Severe	NH
3		M	7.1	L	Conductive	Moderate	NH
4		M	15	R	Conductive	Moderate-to-severe	NH
5		M	12.7	R	Mixed	Severe	NH
6		F	11.1	L	Conductive	Severe	NH
7		M	16.5	L	Conductive	Moderate-to-severe	NH
8		M	8.3	R	Conductive	Severe	NH
9		M	17.1	L	Conductive	Moderate	NH

**Abbreviations:** F, female; HL, hearing loss; L, left; M, male; NH, normal hearing; R, right.

**Table 2:** Surgical and postoperative soft-tissue outcomes

Subject		Surgery time (min)	Surgical technique	Anesthesia	Implant length (mm)	Abutment length (mm)	Postoperative follow-up period (m)	Holgers score
1		20	MIPS	L	4	9	21	0
2		20	MIPS	L	4	6	4	0
3		30	MIPS	L	4	9	15	1
4		40	MIPS	L	4	12	5	3*
5		30	MIPS	L	4	9	4	0
6		27	MIPS	L	4	12	15	1
7		20	MIPS	L	4	9	6	1
8		60	ILTP	L + S	3	9	18	2*
9		29	MIPS	L	4	9	15	1

**Abbreviations:** ILTP, linear incision with tissue preservation; L, local anesthesia; L + S, local anesthesia + sedation; m, months; min, minutes; MIPS, minimally invasive Ponto Surgery; mm, millimeters.

**Note:** *Adverse skin reaction (Holgers score ≥ 2).

One subject (#S8) showed implant loss ∼ 13 months after surgery due to trauma. For the remaining subjects, implant extrusion was not observed, and revision surgery was not required in the entire follow-up period.

**Fig. 3:**
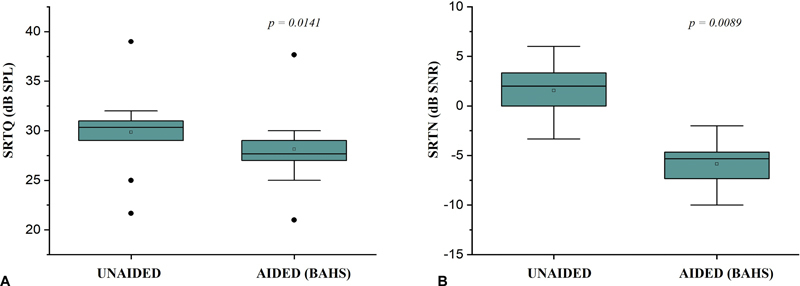
Comparison of the mean scores for speech recognition threshold in quiet ( **A** ) and speech recognition threshold in noise ( **B** ) obtained from subjects between unaided and aided (bone-anchored hearing system, BAHS) conditions, respectively. The Wilcoxon signed-rank test showed that the thresholds significantly decreased with the use of BAHS, *p* < 0.05, effect size (r) > 0.8.

Fig. 3A,B show the comparison between SRTQ and SRTN mean scores obtained from subjects in the unaided and aided (BAHS) conditions, respectively. The SRTQ and SRTN scores significantly decreased with the use of BAHS, although 1 subject (#S5) showed no changes in the SRTQ comparing both the unaided and aided conditions ( Table 3 ). The mean decrease in the SRTQ and SRTN scores with the use of the BAHS were 1.7 dB and −7.4 dB SNR, respectively.

**Table 3:** Comparison of the sentence recognition threshold in quiet and in noise between unaided and aided (BAHS) conditions across subjects

	SRTQ (dB SPL)		SRTN (dB SNR)	
Subject	Speech: front (0°) Quiet		Speech: impaired ear, Noise: NH (S90-N90)	
	Unaided	Ponto	Unaided	Ponto
1	21.6	21	-2	-10
2	31	27	4	-2
3	32	27.6	0	-6.66
4	39	37.6	2	-5.33
5	25	25	-3.33	-9.33
6	29	27.6	1.33	-5.33
7	29.6	29	2.66	-7.33
8	30.3	28.3	3.33	-4.66
9	31	30	6	-2

**Abbreviations:** dB SNR, decibel signal-to-noise ratio; dB SPL, decibel sound pressure levels; NH, normal hearing; Ponto, with the use of the bone-anchored hearing system; SRTN, speech recognition threshold in noise; SRTQ, speech recognition threshold in quiet; Unaided, without the use of the bone-anchored hearing system.

**Fig. 4:**
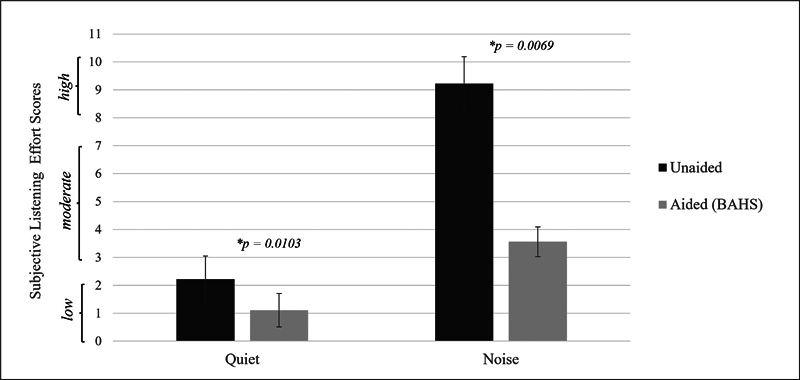
Comparison of the perceptual listening effort scores of the subjects in the speech tasks in quiet and noise, between unaided and aided (bone-anchored hearing system, BAHS) conditions. The Wilcoxon signed-rank test showed a significant decrease of the perceptual listening effort scores with the use of BAHS, *p* < 0.05, effect size (r) > 0.8.

The perceptual listening effort scored by the subjects was much higher in the speech perception-in-noise task than in quiet. It significantly decreased with the use of BAHS in both situations. The listening effort category perceived by the subjects decreased from high to moderate with the use of BAHS in the speech-in-noise task ( Fig. 4 ). The mean SADL global score of the subjects was of 6.0 ± 0.3, indicating that they were very satisfied with the device. Still, the daily use of the BAHS by the subjects ranged from 6 to 11.5 (mean: 8.2 ± 2.0) hours per day.

## Discussion

The purpose of this study was to investigate the hearing performance and soft tissue outcomes of the MIPS under local anesthesia in children with unilateral craniofacial malformation implanted with percutaneous BAHS. It was possible to install the implant in 8 of 9 subjects using this procedure with no intraoperative complications, thereby showing to be a viable option for use in the clinical routine.

Adverse skin reactions were minimal, and it occurred only in one subject (11.1%) during the entire postoperative follow-up period, considering those who had their implant installed with MIPS. The soft-tissue outcomes found in this study are consistent with a retrospective study 7 on 14 pediatric patients who underwent a MIPS procedure to install the BAHS implant, and also with those reported for adults with this procedure. 6 Based on this, we assume that the soft-tissue outcomes reported with the use of MIPS in adults seem to be replicable children, that is, the use of MIPS to install the BAHS implant should not increase the risk of postoperative soft-tissue complications in pediatric patients. Furthermore, in our study, all subjects underwent the MIPS procedure under local anesthesia and then, the effects of their exposure to the general anesthesia were reduced, thereby providing an additional benefit for this population.

The mean surgical time was of 30.7 ± 12.78 minutes, and it comprised the entire time of the surgical procedure, including preparation and aftercare. Our results are comparable with those found by French et al. 7 since they reported a combined average of 12.4 ± 2.6 minutes for skin-to-skin surgical time. Still, even considering the entire time of the surgical procedure in our study, we found shorter surgical time with MIPS than those reported by the authors for Baha Attract (56 minutes) and Connect (53 minutes) (Cochlear Baha Products and Services, Mölnlycke, Sweden) procedures in pediatric patients. 7

Implant loss occurred only in 1 patient (#S8), who had the implant installed by LITP, due to trauma. It is known that one of the risk factors for implant loss in children is the higher incidence of head trauma. 4 14 Considering MIPS, although children with craniofacial malformation have a higher risk of implant loss due to the bone abnormalities and problems with peri-implant hygiene on those with underlying syndromes, 4 osseointegration did not fail for any subject during the mean follow-up period of 11.4 months.

We found significant improvements in the auditory performance of the subjects in quiet and in noise with BAHS. A decrease in the SRTQ and SRTN scores in the aided (BAHS) condition reflected on lower sentence recognition thresholds with the device and indicated a positive outcome. The mean improvement was stronger in noise compared with in quiet, and all subjects included in our study showed lower speech recognition thresholds in noise with the use of BAHS compared with unaided condition. It is known that speech recognition performance in noise can be problematic for patients with unilateral hearing loss, particularly when the noise source is positioned near the better ear and the speech stimulus is presented at or near the poorer ear, with this being the most challenging situation for them. Then, wearing a BAHS on the affected side would improve that situation, 15 and the best speech perception scores in the aided (BAHS) situation would then be expected. Willenborg et al. 16 found a significant improvement of −2.1 dB SNR in speech perception in noise, in the S0N90 situation (speech coming from the front and noise applied to the normal hearing ear), with the use of active osteointegrated implants in 6 adult patients with unilateral hearing loss. They found an even stronger improvement in speech perception of −4.2 dB SNR, when the patients were evaluated in the S90N90 situation. The authors mentioned that the results could be even better with the use of the device in the last test situation (S90N90), but this was the last test performed, and fatigue and a lack of concentration might have led to a decrement in their performance. In our study, we found a significant mean improvement in speech perception in noise of −7.2 dB SNR with the use of BAHS in children with unilateral craniofacial malformation in the S90N90 test situation. Considering that this was the second test performed and no fatigue occurred, stronger improvements could be expected when comparing our results to those found by Willenborg et al. 16 A previous study showed that, for speech-in-noise tests, 1dB decrease in S/N ratio might result in up to 15% better speech recognition. 15 Based on this, our results suggest a significant improvement in auditory performance of these patients with the use of BAHS in their most challenging daily life situation. 17

In quiet, although the mean scores significantly decreased with the use of the BAHS (mean improvement of 1.7 dB SPL), 1 subject showed equal SRTQ when comparing unaided and aided (BAHS) conditions. Kunst et al. 18 found significant improvements in the speech perception scores of adult patients with UCMs implanted with BAHS, but some patients showed equal scores when comparing unaided and aided (BAHS) conditions. Children were not tested in quiet in their study.

The perceptual listening effort reported by the subjects was higher in the speech-in-noise test, and it significantly decreased with the use of BAHS in both the quiet and noise conditions. However, the listening effort category perceived by the subjects decreased with BAHS in the noise condition while it remained equal in quiet, suggesting that the device was more helpful in the most demanding listening situation to them. Listening effort was defined as the deliberate allocation of mental resources to overcome obstacles in goal pursuit when carrying out a listening task. 19 The study by Hornsby 20 showed that individuals with hearing loss experience greater effort than individuals with normal hearing to complete intelligibility tests. Lunner et al. 21 investigated the listening effort through the ability to recall words in patients with BAHS connected to both, abutment and soft band, and they found that listeners' recall ability increased when the sound was directly transmitted to the cochlea via abutment. Bianchi et al. 22 investigated the listening effort via pupillary responses in 21 adult BAHS users with conductive or mixed conductive-sensorineural hearing loss. They showed that the overall effort to process a moderate-to-loud speech signal was significantly reduced by using a sound processor with a higher maximum force output (MFO), due to fewer saturation artifacts. In our study, all subjects used a SuperPower sound processor (Oticon Medical), with a high MFO of 135 dBµN, which may have reflected in the significant reduction of the subjective listening effort, including in the speech test in quiet.

The subjects were very satisfied with the device in daily life. High levels of satisfaction and increased quality of life with BAHS were previously reported. 23 24 The subjects were using their BAHS daily for more than 8.2 hours, on average, showing high compliance with the device use. It is known that long daily use of the device suggests patient satisfaction, 25 and these results are comparable with those found in the SADL questionnaire.

## Conclusion

Minimally invasive Ponto surgery under local anesthesia showed to be a viable option for percutaneous BAHS implant installation in children with UCM. The hearing performance of the subjects improved with BAHS, and they were globally satisfied with the device, using it for more than 8 hours a day, on average. Soft-tissue complications were minimal, and our results are comparable to those reported in the literature regarding MIPS in adults.
